# Case of Acute Rheumatism, Cured by Bandaging

**Published:** 1816-01

**Authors:** H. Ronalds

**Affiliations:** Member of the Royal Medical Society of Edinburgh, and of the Royal College of Surgeons in London.


### *For the London Medical and Physical Journal.*

*Case of Acute Rheumatism, cured by Bandaging;* by H. Ronalds, Member of the Royal Medical Society of Edinburgh, and of the Royal College of Surgeons in London.

DR. BALFOUR has, in the 42d number of the Edinburgh Medical and Surgical Journal, adduced a variety of cases to prove the efficacy of bandages in chronic rheumatism. As the evidences in favour of a newly-proposed remedy cannot be too strong, I send the following case: in hopes it will afford additional interest, as it shews the decidedly good effects of the above method of practice in another form of the disease, viz. the acute species.

Mrs. ——, a lady of delicate constitution, requested my attendance on the 13th October, and related to me the following symptoms. She complains of a general sense of soreness, particularly in the thighs and knees; indisposition to use the least exertion; and the slightest motion of the body occasions pain in the larger joints, particularly in the hips and knees; the latter, at present, not at all swollen or inflamed. She has likewise nausea; the tongue is foul; thirst urgent; bowels rather costive; pulse 98, rather weak; heat increased; skin dry; sleep much disturbed.

The above symptoms have existed for nearly four days. Languor, general debility, accompanied by disordered stomach, preceded the febrile symptoms; and the latter, together with general pains, she thinks have been more urgent this day and yesterday. She imputes her illness to a cold received while standing in a wet kitchen; has taken no other remedies than a dose of calomel.

℞. Pulver. Rhei ℈j.

Potass. Tart. ʒij.

Tinct. Rhei Comp. f ʒifs.

Aquæ Carui f. ℥ifs. fiat haustus aperiens quamprim. sumend. etiamque fricentur genu et coxa Linimento Ammoniæ Carbonatis, et habeat horâ somni Pulver. Ipecac. Comp. gr. xij.

14th.—A restless night; skin dry; pains greatly increased in the hips and knees; the latter swollen considerably; pulse 100, rather feeble.

℞. Liquor. Ammon. Acet. f ℥ij.

Confect. Opii ℈ij.

Liquor. Antim. Tart. f ʒifs.

Aq. Menthæ f ℥vfs.

Spir. Ammon. Arom. f ʒij. misce, capiat cochliaria duo tertiâ quaque horâ.

Continuatur Pulvis Ipec. Comp.

15th.

15th.—A sleepless night; pains excruciating; knees and ankles considerably swollen; pulse 100, still small; tongue foul; skin rather moist.

℞. Pulv. Ipecac. gr. xv.
Vini Ipecac. f ℥vj. pro emetico hoc vesperi sumendus.
Postea repetatur mistura salina, et habeat horâ somni
Pulveris doctor. Jacobi, gr. vj.
Nitratis Potass, gr. v.

16th.—Symptoms nearly as yesterday; skin now moist, with perspiration. Repetantur mistura et Pulvis Jacobi.

℞. Pulver. Ipecac. Comp. ℨſs.

Confect. Rosæ Gall. q. s. ut fiant pilulæ decem; quarum sumat duas quarta quâque horâ.

17th.—The pains, if possible, increased; she is totally incapable of moving without assistance; swelling of the knees continues; ankles inflamed, and much swollen. While visiting my patient to-day, I observed that her hands were constantly applied to the most pained part (the left knee), and she thought the pressure afforded some relief. The idea of bandaging immediately occurred to me, although, from the very acute form of the disorder, I had hitherto judged it an improper case for the roller. A bandage of about three yards in length was, however, now applied to both limbs, from the foot to the middle of the thigh. The relief obtained was instantaneous; indeed pain no longer existed; she bent the knees, and moved both limbs with freedom. From that moment I considered my patient to be convalescent, and am happy to say that my expectations are completely realised, for the febrile and other symptoms have entirely disappeared, and she is now a stranger to pain in any part.

*Oct.* 25, 1815.

